# Bacillus anthracis strain differentiation based on SNP and VNTR loci

**DOI:** 10.18699/VJGB-22-68

**Published:** 2022-10

**Authors:** E.A. Anisimova, N.A. Fakhrutdinov, D.A. Mirgazov, E.A. Dodonova, I.A. Elizarova, M.E. Gorbunova, N.I. Khammadov, L.I. Zainullin, K.A. Osyanin

**Affiliations:** Federal Center for Toxicological, Radiation and Biological Safety, Nauchny Gorodok-2, Kazan, Russia; Federal Center for Toxicological, Radiation and Biological Safety, Nauchny Gorodok-2, Kazan, Russia; Federal Center for Toxicological, Radiation and Biological Safety, Nauchny Gorodok-2, Kazan, Russia; Federal Center for Toxicological, Radiation and Biological Safety, Nauchny Gorodok-2, Kazan, Russia; Federal Center for Toxicological, Radiation and Biological Safety, Nauchny Gorodok-2, Kazan, Russia; Federal Center for Toxicological, Radiation and Biological Safety, Nauchny Gorodok-2, Kazan, Russia; Federal Center for Toxicological, Radiation and Biological Safety, Nauchny Gorodok-2, Kazan, Russia; Federal Center for Toxicological, Radiation and Biological Safety, Nauchny Gorodok-2, Kazan, Russia; Federal Center for Toxicological, Radiation and Biological Safety, Nauchny Gorodok-2, Kazan, Russia

**Keywords:** Bacillus anthracis, genotyping, VNTR, SNP, HRM-analysis, epidemiology of anthrax, Bacillus anthracis, генотипирование, VNTR, SNP, HRM-анализ, эпидемиология сибирской язвы

## Abstract

Bacillus anthracis is the anthrax causative agent. For its epidemiology, it is important not only to identify the etiological agent but also to determine the patterns of its evolution and spread. Modern methods of molecular biology make it possible to detect a number of genetic markers suitable for indicating and differentiating the strains of B. anthracis, including the loci arranged as variable number tandem repeats (VNTRs) and SNPs, one nucleotide-sized differences in the DNA sequence of the loci being compared. The objective of the present study was to examine the effectiveness of SNP analysis and PCR amplif ication of VNTR loci combined with the high-resolution amplicon melting analysis for identif ication and differentiation of the anthrax agent strains. In the study, seven strains of B. anthracis obtained from soil samples and animal carcasses were investigated using vaccine strain STI-1 as a reference. For molecular genetic characterization of these bacteria, analysis of 12 SNPs and variability analysis of eight VNTR loci were carried out. To detect the differences between the strains, their PCR product melting points were measured in the presence of the EvaGreen (Sintol, Russia) intercalating dye. For SNP detection, a PCR assay with double TaqMan probes was applied. It was found that the studied virulent strains, except for B. anthracis No. 1 and 3, could not be attributed to any phylogenetic subgroup of the anthrax agents. The proposed method made it possible to differentiate four out of the seven investigated strains. Strains No. 5–7 had identical SNP and HRM prof iles and, as a result, formed a single cluster. Our investigation has conf irmed that the proposed method can be successfully used for preliminary analysis of an epizootic situation in the case of anthrax.

## Abstract (ru)

Бактерии Bacillus anthracis являются возбудителем сибирской язвы. Для эпидемиологии этой инфекции имеет значение не только идентификация этиологического агента, но и выяснение закономерности его эволюции и распространения. Современные методы молекулярной биологии позволяют определить ряд генетических маркеров, пригодных для индикации и дифференциации штаммов B. anthracis. К таким маркерам относят VNTR-локусы – последовательности, организованные в геноме в виде тандемных повторов, а также SNP – отличия в последовательности ДНК в сравниваемых локусах размером в один нуклеотид. Целью настоящей работы была оценка эффективности совместного применения SNP-анализа и ПЦР-амплификации VNTR-локусов с анализом температуры плавления ампликонов высокого разрешения для идентификации и дифференциации штаммов возбудителя сибирской язвы. Исследовали семь штаммов B. anthracis, полученных из образцов почвы и трупов животных, в качестве референс-микроорганизма был вакцинный штамм B. anthracis СТИ-1. Для молекулярно-генетической характеристики данных бактерий проведен анализ 12 однонуклеотидных полиморфизмов, а также вариабельности восьми VNTR-локусов, для определения различий в которых был впервые использован метод определения температур плавления ПЦР-продуктов в присутствии интеркалирующего красителя EvaGreen (ЗАО «Синтол», Россия). Для детекции SNP применен метод полимеразной цепной реакции (ПЦР) с использованием двойных TaqMan-зондов. Обнаружено, что все изучаемые вирулентные штаммы, кроме B. anthracis № 1 и 3, по SNP-профилю не могут быть отнесены к какой-либо филогенетической подгруппе возбудителя сибирской язвы. Методический подход, включающий в себя анализ SNP- и VNTR-последовательностей, позволил диффе- ренцировать между собой штаммы B. anthracis № 1–4, в то время как бактерии B. anthracis № 5–7 демонстрируют одинаковые SNP- и HRM-профили и, как следствие, формируют один кластер. Таким образом, показана принци- пиальная возможность использования рассмотренной в этой работе методики для предварительного анализа эпизоотической ситуации при вспышках сибирской язвы.

## Introduction

Bacillus anthracis is the causative agent of anthrax, a particularly hazardous zoonotic infection. Although effective measures to prevent the occurrence and spread of the disease have been developed and rather widely implemented, from 2000 to 20,000 anthrax cases are registered around the world every year (Pisarenko et al., 2019), mostly in Africa, Central Asia, and Latin America (Hugh-Jones, Blackburn, 2009; Kenefic et al., 2009). In Russia, anthrax commonly occurs in Siberia and North Caucasus (Logvin et al., 2017).

All the B. anthracis populations known to researchers are extremely monomorphic and have clonal structure (Achtman, 2008; Keim et al., 2009). This high genetic similarity poses a significant hindrance for strain differentiation of anthrax agents using bacteriological and serological methods. The problem, however, may be solved using molecular genetic approaches. The methods detecting sites with variable number tandem repeats (VNTR) and single nucleotide polymorphisms (SNP) in the agent’s genome have turned out to be the most promising for B. anthracis strain indication and differentiation (Timofeev et al., 2018; Wang et al., 2020).

Compared to VNTR loci, SNPs are more stable in evolutionary perspective and have low mutation frequency, which comes at a cost of lower resolution. That is why polymorphism detection in SNP loci of the anthrax agent is a rather common first stage in the genotyping systems using a combination of SNP and VNTR markers (Timofeev et al., 2018), in which a set of 14 diagnostically significant canonical SNPs (canSNPs) is widely used. The systems make it possible to attribute the microorganism under study to a particular phylogenetic line and, as a result, make an assumption about its geographic origin (Van Ert et al., 2007). Three phylogenetic lines (A, B, and C) are commonly identified in today’s research, which in turn form 14 phylogenetic groups as follows: A.Br.Ames, A.Br.Australia 94, A.Br.003/004, A.Br.Vollum, A.Br.005/006, A.Br.001/002, A.Br.Western, A.Br.WNA, A.Br.008/009, A.Br.011/009, B.Br.001/002, B.Br.KrugerB, B.Br.CNEVA, and C.Br.A1055 (Timofeev et al., 2018). According to the literature, the strains isolated in the Russian Federation belong predominantly to group B.Br.001/002 of line B and groups A.Br.001/002 and A.Br.008/009 of line A, and less often to A.BrAust94 (Eremenko et al., 2018; Koteneva et al., 2019).

Multilocus variable number tandem repeat analysis (MLVA) is used for further strain differentiation within each SNP cluster (Timofeev et al., 2018). PCR analysis with further separation of amplification products in agarose or polyacrylamide gel, often combined with capillary electrophoresis, is the most common MLVA strategy (Bondareva et al., 2014). The most accurate results may be obtained from amplicon sequencing, but the duration of the procedure (at least several days) tends to be a major downside of the approach.

In the present study, differences in VNTR loci, specifically tandem repeat numbers, were determined using HRM (high resolution melting), i. e. real-time analysis of amplicon melting points. The EvaGreen (Sintol, Russia) intercalating dye used for HRM analysis inserts itself between two complementary nucleotides in a double-stranded DNA molecule. The dye’s fluorescence under light of 490-nm wavelength is registered in FAM detection channel. When DNA denaturation occurs, there is no fluorescence and hydrogen bonds break. Thus, if we gradually increase the temperature in the thermocycler, continuous detection will enable us to determine the repeat number based on amplicon melting point. The latter approach outperforms the classical MLVA methods since it does not require sequencing or fluorescence-labeled probes, and, as a result, makes it possible to detect differences in VNTR loci of B. anthracis strains at a lower financial and time cost. HRM analysis of PCR amplification products had been previously suggested for SNP genotyping (Derzelle et al., 2011) but had never been described for analyzing VNTR loci.

The objective of the present study was to evaluate the efficiency of SNP analysis and PCR amplification of VNTR loci in combination with high-resolution amplicon melting point analysis for identification and differentiation of anthrax agent strains.

## Materials and methods

In the study, seven B. anthracis strains obtained from soil samples and animal carcasses were used (Table 1). B. anthracis vaccine strain STI-1 from the microorganism strain collection of the Federal Center for Toxicological, Radiation and Biological Safety (Kazan, Russia) was used as a reference. The strain samples were prepared for further molecular genetic research in compliance with the MUK 4.2.2941-11 methodological protocol (2011).

**Table 1.**
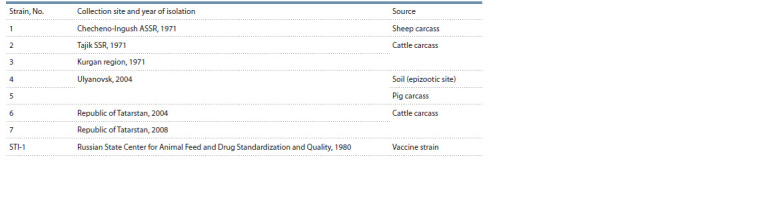
Studied strains of B. anthracis

The genomic DNA was isolated using the DNA-sorb-B kit (Central Research Institute for Epidemiology of Rospotrebnadzor, Moscow) as per manufacturer’s instructions.

The SNP analysis was performed using real-time polymerase chain reaction (qPCR) with the double TaqMan probes and primers described earlier (Van Ert et al., 2007). The master mix volume of 15 μl included 125 μM of each dNTP, 2.5 мМ MgCl2, 5 pM of each primer and probe, 10 ng DNA matrix, 1.0 U Taq polymerase (Evrogen, Russia), ddH2O (up to 15 μl). The qPCR procedure was run on a Real-Time С1000 thermocycler with CFX96 optical reaction module (Bio-Rad, USA) under the following protocol: initial DNA denaturation at 95 °C for 3 minutes followed by 39 cycles as follows: denaturation at 95 °C for 10 s, annealing oligonucleotides at 50 °C for 30 s (detection in R6G/ROX channel), extension at 72 °C for 5 s. Point nucleotide changes in each locus were identified based on fluorescence intensity in each channel. Variability of SNP loci was numerically estimated based on allelic polymorphism index (h) (Selander et al., 1986).

MLVA amplicon melting points were determined using the EvaGreen intercalating dye. The primer set for amplification of VNTR loci is presented in Table 2.

**Table 2.**
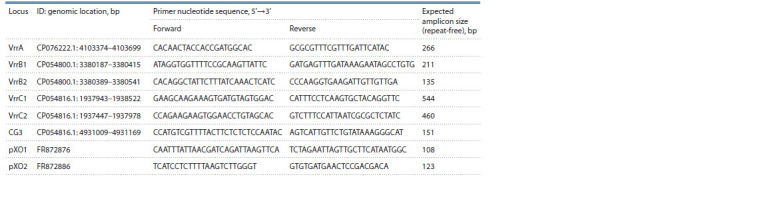
Synthetic oligonucleotides used for amplification of VNTR loci

The qPCR master mix volume of 15 μl included 1.5 μl 10 × PCR-buffer with the EvaGreen dye (Syntol), 2.5 мМ MgCl2 solution (Syntol), 1.0 U Taq polymerase (Syntol), 125 μM of each dNTP, 5 pM forward and reverse primers, 10 ng DNA matrix, ddH2O (up to 15 μl). DNA amplification with further HRM analysis was run on a Real-Time С1000 thermocycler with CFX96 optical reaction module (Bio-Rad) under the following protocol: initial DNA denaturation at 95 °C for 3 min followed by 39 cycles as follows: denaturation at 95 °C for 10 s, annealing oligonucleotides at 60 °C for 30 s (detection in FAM channel), extension at 72 °C for 10 s. Melting parameters were as follows: temperature range from 65 to 95 °C with 0.2 °C increments, 5 s dwell time. Melting curves for amplification products were graphically analyzed using CFX ManagerTM (Bio-Rad). The amplicons were separated in native 8 % polyacrylamide gel (PAAG) (Sambrook et al., 1989).

Bioinformation analysis of B. anthracis genomes was performed using the Vector NTI 9.1 software and NCBI databases (https://www.ncbi.gov).

## Results

Design and validation

Molecular typing of the strains was performed using the extended protocol including the detection of 12 SNP loci referred to as A.Br.001, A.Br.003, A.Br.004, A.Br.006, A.Br.007, A.Br.008, A.Br.009, B.Br.001, B.Br.002, B.Br.003, B.Br.004, and A/B.Br.001 (Van Ert et al., 2007) and analysis of eight VNTR loci (VrrA, VrrB1, VrrB2, VrrC1, VrrC2, CG3, pX01, and рХ02) (Keim et al., 2000). The genotyping was validated using B. anthracis vaccine strain STI-1.

The results of SNP analysis presented in Table 3 allowed us to draw conclusions on the configuration of point nucleotide changes in the 12 investigated loci of B. anthracis STI-1. It was found that, except for two loci (ABr003 and ABr008), the SNP profile obtained matched the data on single nucleotide polymorphisms available in the literature for the same strain (Afanas’ev et al., 2014; Eremenko et al., 2018). It should be noted that canSNPs are rather conservative and known for low mutation rate (Timofeev et al., 2018). Therefore, the validity of atypical single nucleotide changes in loci ABr003 and ABr008 detected for B. anthracis STI-1 required further confirmation, particularly by sequencing. Thus, ABr003 and ABr008 loci were discarded from the canSNP panel applicable for strain differentiation at the current research stage

**Table 3.**
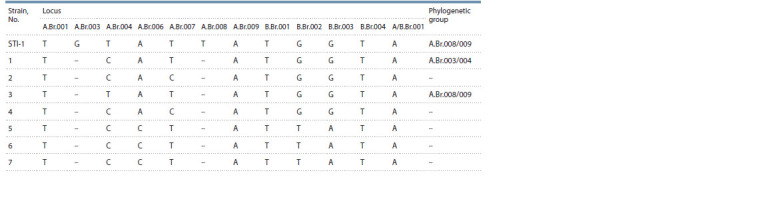
Results of SNP analysis of investigated B. anthracis strains

MLVA was performed using classical PCR with further amplicon separation in PAAG that only allowed us to determine the approximate sizes of seven investigated VNTR loci of B. anthracis STI-1 as follows: VrrA – 300 bp, VrrB1 – 250 bp, VrrB2 – 190 bp, VrrC1 – 700 bp, VrrC2 – 600 bp, CG3 – 160 bp, pX01 – 160 bp. Amplification products for the locus localized in capsule-encoding plasmid pX02 were not detected. The absence of plasmid pXO2 is characteristic for B. anthracis STI-1 (Afanas’ev et al., 2014).

The accurate size of the amplified VNTR fragments of the B. anthracis STI-1 DNA as well as nucleotide repeat sizes were determined in silico. Chromosomal DNA nucleotide sequence of B. anthracis STI-1 (GenBank CP066168) was limited by the respective primers (see Table 2) using the Vector NTI 9.1 software. The results obtained after bioinformation analysis of the B. anthracis STI-1 genome are presented in Table 4. The complete nucleotide sequence of plasmid pX01 for the investigated strain is not available in the GenBank database. As a result, the tandem repeat number in the locus of interest was determined as a difference between the amplicon molecular mass and the repeat-free size of the amplified fragment divided by the number of nucleotides in the variable site. Electrophoresis showed that CG3 and pX01 locus sizes for B. anthracis STI-1 were identical. Thus, to calculate the repeat number for plasmid pX01, the molecular mass of the CG3 locus was used.

**Table 4.**
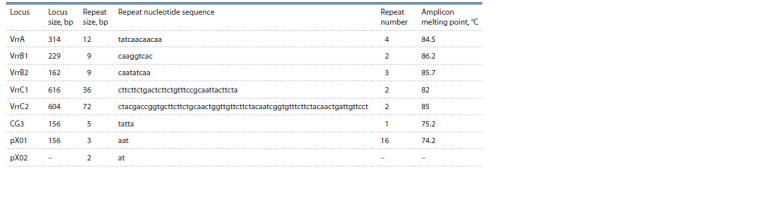
VNTR locus characteristics of B. anthracis STI-1

The melting curve peaks for qPCR amplification products for MLVA loci of B. anthracis STI-1 were also identified (see Table 4). The qPCR procedure was performed using the EvaGreen dye in eight replicas. It was found that the difference between replicas for most loci under study was 0.2 °С. Thus, the values of at least 0.2 °С were considered an acceptable parameter difference for the further differentiation of anthrax strains based on VNTR loci melting point differences.

Single nucleotide polymorphism analysis

The results of molecular genetic analysis of SNP loci for the studied virulent strains of B. anthracis are shown in Table 2. It turned out that SNP analysis did not show differences in five loci including A.Br001, A.Br005, A.Br009, B.Br001, A/B. Br001. The values of variability index (h) for the remaining seven SNP loci ranged from 0.12 to 0.4.

The results obtained allowed us to divide the investigated strains into four clusters. The largest cluster was formed by the three strains isolated in the Republic of Tatarstan (B. anthracis No. 6 and 7) in 2008 and 2014 and in Ulyanovsk (B. anthracis No. 5) in 2004. The second cluster included strains No. 2 and 4 collected in the Tajik SSR in 1972 and Ulyanovsk in 2004, respectively. Two remaining clusters were formed by strains No. 1 and 3 found in the Checheno-Ingush ASSR in 1971 and in the Kurgan region in 1972, respectively

The results of SNP typing allowed us to attribute strain No. 1 to the phylogenetic subgroup A.Br.003/004. It was found that B. anthracis bacteria of strain No. 3 could be attributed to the phylogenetic line A.Br.011/009, similarly to the STI-1 reference strain. The SNP profiles obtained for the rest of the investigated microorganisms were not characteristic for any of the previously identified phylogenetic subgroups of B. anthracis strains.

Multilocus variable number tandem repeat analysis

The obtained amplicon melting point values were used to perform VNTR strain differentiation (Table 5). The melting points of the obtained PCR fragments after variable-locus amplification depended on their nucleotide compositions, specifically the tandem repeat numbers, i. e., the higher the latter, the higher the melting point.

**Table 5.**
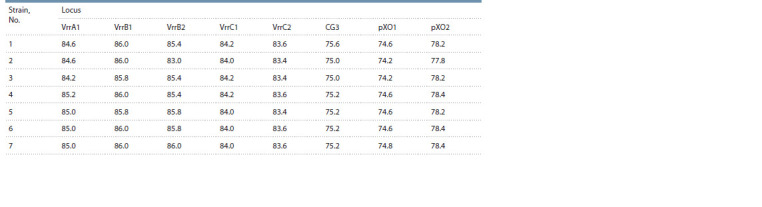
Melting points of the PCR products obtained after amplification in the VNTR loci of the investigated B. anthracis strains

It was found that the melting curves of strains No. 1 and 2 obtained after amplification in the VrrA1 locus were identical to the melting curve obtained for the reference strain at the same locus and probably included four tandem repeats in VrrA1. Four strains (No. 4–7) showed higher melting points compared to the reference strain, which implies a higher tandem repeat number in the VrrA1 locus. On top of that, strain No. 3 showed the lowest melting point in the VrrA1 locus among the investigated strains, and as a result it was characterized by the lowest repeat number in the VrrA1 locus.

Strains No. 5–7 and STI-1 showed identical amplicon melting curves at VrrB2 and, thus, included three tandem repeats in the VrrB2 locus, similarly to B. anthracis STI-1. The data from Table 5 demonstrate that the rest of the investigated strains had lower repeat numbers in this locus, i. e., probably two in strains No. 1, 3, 4, 6 and one – in strain No. 2.

The amplicon melting curves in the CG3 locus indicate that all the strains of interest, except for No. 1, included one tandem repeat, similarly to STI-1, so strain No. 1 probably carries several repeats in this locus.

It was observed that strains No. 2 and 3 were characterized by amplicon temperatures in pX01 identical to those of the reference strain. Thus, a conclusion was made that these bacteria had 16 tandem repeats in plasmid pX01, whereas the remaining strains had over 16 repeats.

The melting points of PCR products obtained after amplification in loci VrrB1, VrrC1, and VrrC2 were the same for all investigated strains, and, as a result, their repeat numbers fragments were the same in these DNAs as well. STI-1 had two tandem repeats in these loci. The investigated strains showed lower amplicon melting points in VrrB1 and VrrC2 compared to the reference strain and probably carried one tandem repeat in these loci. With regards to the VrrC1 locus, the melting points of PCR products obtained after amplification for all the investigated strains exceeded the respective value of the reference strain. Thus, a conclusion can be made that the investigated strains carry three or more tandem repeats in the VrrC1 locus. The number of repeats in the pX02 locus could not be determined due to the lack of this marker in the reference strain.

Thus, it was found that strain No. 1 had a different tandem repeat number in the CG3 locus, strain No. 2 – in the VrrB2 and pXO2 loci, and strain No. 3 – in the VrrA1 locus. A unique melting point profile of PCR amplification products in VNTR loci was identified for strains No. 1–3, making it possible to differentiate between them. The remaining three strains (No. 5–7) showed the same melting point profiles and could therefore be combined into one cluster. A similar melting point profile was detected for strain No. 4, its only difference from strains No. 5–7 being the tandem repeat number in the VrrB2 locus.

## Discussion

In recent years, Russian and foreign authors have published a substantial number of papers on the design of viable approaches to genotyping anthrax agent strains (Le Flèche et al., 2001; Keim et al., 2004; Van Ert et al., 2007; Gierczynski et al., 2009; Eremenko et al., 2012; Afanas’ev et al., 2014). Most of the available B. anthracis genotyping methods are based on polymorphism of tandem repeats or point mutations in their genome. Some authors believe that the test systems based on a combination of genetic markers with different discriminating power and stability are the most efficient way to differentiate between B. anthracis strains (Keim et al., 2004; Chang et al., 2007; Afanas’ev et al., 2014). SNP loci are more stable but have a low variability index compared to VNTR ones. That is why, when these loci are used in combination, it is recommended to first use canSNPs to attribute the investigated strains to a specific phylogenetic group, and then use MLVA to differentiate the strains within each canSNP cluster (Timofeev et al., 2018).

PCR amplification followed by visualization of the obtained amplicons in agarose or polyacrylamide gel is still considered a universal approach to the analysis of VNTR loci (Jackson et al., 1997, 1999; Keim et al., 2000). This method may be effective for differentiation of nucleotide repeats of over 10 bp in size (VrrA1 – 12 bp, VrrC1 – 36 bp, VrrC2 – 72 bp) but is not suited for differentiation of repeats of 2–3 nucleotides in size (VrrB1 and VrrB2 – 9 bp, CG3 – 5 bp, pX01 – 3 bp, рХ02 – 2 bp). In our study, the sizes of most repeats did not exceed 10 bp, which made electrophoresis unviable for strain differentiation, so HRM analysis was applied to differentiate between the allelic variants of the VNTR loci based on tandem repeat numbers. This method is widely used in genotyping, specifically to detect mutations, polymorphisms, and epigenetic differences in double-stranded DNA samples (Graham et al., 2005; Margraf et al., 2006). According to the literature, HRM analysis is also applicable for indication and differentiation of Brucella strains (Winchell et al., 2010)

According to the literature, the B. anthracis strains circulating in the Russian territories of high anthrax risk are predominantly attributed to the A.Br.001/002, A.Br.008/009, B.Br.001/002, and A.BrAust94 genotypes (Eremenko et al., 2018; Kravets et al., 2018; Koteneva et al., 2019). In particular, the strains isolated in North Caucasus generally fall into canSNP clusters A.Br.008/009 and A.BrAust94. Genotype B.Br.001/002 also occurs in the Republic of Dagestan (Koteneva et al., 2019). The strains attributed to phylogenetic groups A.Br.001/002, A.Br.008/009, and B.Br.001/002 are the most common in Siberia and the Russian Far East (Eremenko et al., 2018; Kravets et al., 2018).

It should be noted that, according to some authors, the variety of canSNP genotypes is probably not restricted to the 14 that are currently described (Afanas’ev et al., 2014; Timofeev et al., 2018). For example, M.V. Afanas’ev et al. (2014) have identified three additional phylogenetic subgroups. Indeed, the results of genetic typing of the seven B. anthracis cultures performed in the present study using SNP and MLVA analysis showed that all the studied microorganisms, except for strains No. 1 and 3, could not be attributed to the main phylogenetic canSNP subgroups based on their SNP profiles. Phylogenetic line A.Br.003/004, to which strain No. 1 was attributed, mostly includes strains from the American continents (Eremenko et al., 2018). Among all the investigated strains, strain No. 3 isolated in the Kurgan region showed the most characteristic SNP profile for Russian isolates.

The studied anthrax agent strains are typically organized into SNP clusters based on their geographic origin, the exception being strains No. 2 and 4 isolated in the former Tajik SSR in 1972 and Ulyanovsk in 2004 sharing the same SNP profile. HRM analysis showed that these bacteria had different repeat numbers in loci VrrA1, VrrA2, pXO1, and pXO2 and, as a result, may be differentiated from one another. A reasonable assumption would be that these microorganisms had a common geographic origin but diverged with time affected by trade and migration flows. According to the literature, VNTR loci are characterized by a high mutation rate (10–5 to 10–4 per generation) (Keim et al., 2004; Birdsell et al., 2012; Thierry et al., 2014) and, compared to SNP loci, are in fact the markers of the later evolution of B. anthracis strains.

The HRM profiles obtained for the remaining strains of interest had the patterns matching well with the SNP profiles. Thus, the extended protocol combining SNP and VNTR analyses makes it possible to differentiate between four B. anthracis strains. Strains No. 5–7 demonstrated the same SNP and HRM profiles and were therefore combined into the same cluster

The use of HRM analysis has made it possible to differentiate the strains of interest from one another and attribute them to the respective clusters. We have also determined repeat sizes in the loci, the PCR-product melting points of which were identical to amplicon melting points in the same VNTR loci for the reference strain. The state of the art is that the tandem repeat size may only be accurately determined by sequencing, which means VNTR locus sequencing is to be performed for the strains of interest in the future. We believe that combining the two methods may allow us to create a database of melting curves for VNTR loci, in which the curves will be related to the locus size.

## Conclusion

Applying HRM to analyze PCR products in VNTR loci has a high application value. In particular, this approach may be used for a rapid preliminary differentiation of B. anthracis strains within the same outbreak. To achieve the most efficient and informative indication and differentiation of anthrax agent strains, we propose the following algorithm: 1) attribute the strains of interest to phylogenetic subgroups using SNP typing; 2) differentiate the strains within each SNP cluster using MLVA and HRM analysis; 3) perform MLVA typing for the differentiated strains using classical PCR, electrophoresis, and sequencing. However, further research is required to investigate the capabilities and limits of this genotyping strategy.

## Conflict of interest

The authors declare no conflict of interest.
